# HINT1 neuropathy in Norway: clinical, genetic and functional profiling

**DOI:** 10.1186/s13023-021-01746-z

**Published:** 2021-03-04

**Authors:** Silvia Amor-Barris, Helle Høyer, Lin V. Brauteset, Els De Vriendt, Linda Strand, Albena Jordanova, Geir J. Braathen, Kristien Peeters

**Affiliations:** 1 Molecular Neurogenomics Group, VIB-UAntwerp Center for Molecular Neurology, University of Antwerp, Unversiteitsplein 1, Building V, 2610 Antwerpen, Belgium; 2 Molecular Neurogenomics Group, Department of Biomedical Sciences, University of Antwerp, Antwerpen, Belgium; 3 Department of Medical Genetics, Telemark Hospital Trust, Sykehuset Telemark, Postboks 2900 Kjørbekk, 3710 Skien, Norway; 4 Division Elverum-Hamar, Department of Children and Youth, Innlandet Hospital Trust, Elverum, Norway; 5 Molecular Medicine Center, Department of Medical Chemistry and Biochemistry, Medical University-Sofia, Sofia, Bulgaria

**Keywords:** Peripheral neuropathy, Charcot-Marie-Tooth disease, *HINT1*, Neuromyotonia, Norway

## Abstract

**Background:**

Autosomal recessive axonal neuropathy with neuromyotonia has been linked to loss of functional HINT1. The disease is particularly prevalent in Central and South-East Europe, Turkey and Russia due to the high carrier frequency of the c.110G > C (p.Arg37Pro) founder variant.

**Results:**

In a cohort of 748 Norwegian patients with suspected peripheral neuropathy, we identified two seemingly unrelated individuals, compound heterozygous for a new variant (c.284G > A, p.Arg95Gln) and the most common pathogenic founder variant (c.110G > C, p.Arg37Pro) in the *HINT1* gene. Probands presented with motor greater than sensory neuropathy of various onset, accompanied by muscle stiffness and cramps in the limbs. Furthermore, they displayed non-classical symptoms, including pain in the extremities and signs of central nervous system involvement. Haplotype analysis in both patients revealed a common chromosomal background for p.Arg95Gln; moreover, the variant was identified in Swedish carriers. Functional characterization in *HINT1*-knockout and patient-derived cellular models, and in *HNT1*-knockout yeast, suggested that the new variant is deleterious for the function of HINT1 and provided mechanistic insights allowing patient stratification for future treatment strategies.

**Conclusion:**

Our findings broaden the genetic epidemiology of HINT1-neuropathy and have implications for molecular diagnostics of inherited peripheral neuropathies in Scandinavia.

## Background

Loss-of-function alterations in the histidine triad nucleotide-binding protein 1 (HINT1) are associated with autosomal recessive axonal neuropathy with neuromyotonia (NMAN [MIM: 137200]) [1]. The disease onset is mostly around the age of 10 years. Patients with HINT1-deficiency show slowly progressive motor-greater-than-sensory polyneuropathy leading to lower limb weakness and gait impairment [2]. The sensory involvement develops over time, becoming more pronounced upon disease duration. Notably, 70% of affected individuals present with neuromyotonia, a peripheral nerve hyperexcitability clinically manifesting as spontaneous muscular activity at rest and delayed muscle relaxation after voluntary contraction [1, 2].

Nineteen variants have been causally associated with HINT1-neuropathy in over 100 patients from Europe, North America and Asia [1–5]. So far, there are four proven founder variants in Europe (p.Arg37Pro, p.Cys84Arg, p.His112Asn) [1, 6] and China (p.Cys38Arg) [4]. Among them, p.Arg37Pro is by far the most common, due to its high carrier frequency (1:67–250) in Central and South-East Europe, Russia and Turkey [1, 6]. In Czechia and Russia, HINT1-neuropathy ranks among the most frequent forms of axonal neuropathy [5, 6] while in Bulgaria cases of pseudo-dominant inheritance have been identified (A. Jordanova, unpublished data).

HINT1 is a ubiquitous homodimeric purine phosphoramidase belonging to the histidine-triad (HIT) superfamily, characterized by a conserved HIT motif (His-X-His-X-His-X-X) in the catalytic pocket. Although its endogenous substrate remains unknown, in vitro HINT1 hydrolyzes AMP-linked substrates [7] and acts as a SUMO1-cleaving Cys-protease [8]. HINT1 is also known to regulate transcription factors involved in tumor progression and apoptosis [2]. In the central nervous system (CNS), HINT1 interacts with the μ-opioid receptor regulating its desensitization [2].

NMAN-associated HINT1 alterations cause a loss of (enzymatic) function, because they lead to unstable protein or transcript, or affect key residues in the catalytic cleft [1]. Five variants were initially modeled in a *Saccharomyces cerevisiae* strain deficient for *HNT1,* the yeast *HINT1* orthologue [7]. This strain grows normally under standard cultivation conditions but cannot proliferate under stress (high temperature, alternative carbon source). Genetic complementation with human *HINT1* restores yeast growth indicating an evolutionary conserved function. Notably, none of the tested NMAN-variants could complement the *HNT1* deficiency, confirming the loss-of-function hypothesis [1].

Despite the increasing number of HINT1 patients being diagnosed world-wide, it still remains challenging to assess the pathogenicity of novel variants. Moreover, there is increasing evidence that different disease-causing alterations have differential effects on HINT1 protein stability and function. This has important implications for future therapeutic strategies, as the mutational category will determine a patient’s treatment options. Therefore, functional characterization of novel HINT1 variations benefits both diagnostics and patient stratification. In addition, so far, only a few case reports describe “non-classical” symptoms associated with HINT1-neuropathy [9–11], highlighting the need for further study to expand the clinical spectrum and aid clinicians with differential diagnosis.

Here, we performed the first systematic assessment of HINT1 neuropathy in Norway and describe a potential new founder event in the Scandinavian region.

## Results

**Fig. 1:**
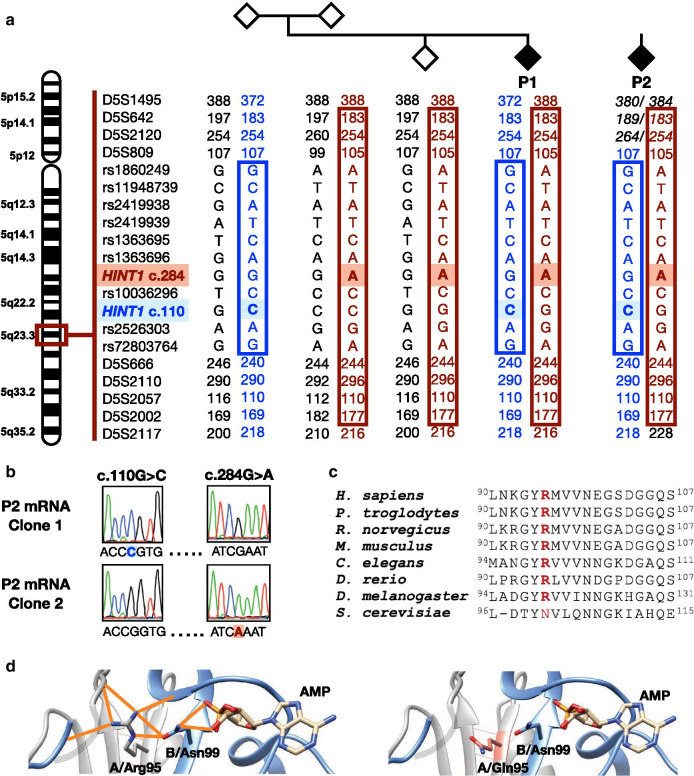
Genetic and in silico characterization of the identified variants in *HINT1*. **a** Pedigree structure, segregation and haplotype analysis. The shared haplotype surrounding the novel c.284G > A (p.Arg95Gln) variant is depicted in red, the previously established haplotype surrounding the known c.110G > C (p.Arg37Pro) founder variant is in blue. **b** Phasing analysis of the two *HINT1* variants found in P2 using cDNA obtained from lymphoblasts. Electropherograms of two individual cDNA clones show that the variants are situated on different transcripts (*in trans*). **c **Evolutionary conservation of the amino acid residue affected by the novel p.Arg95Gln substitution in HINT1. The mutated residue is indicated in red. **d** Left: Localization of the p.Arg95 residue on the crystal structure of HINT1 (PDB:5KLZ) and its interaction with other amino acids through hydrogen bonds (orange). Right: The p.Arg95Gln substitution (in salmon) causes a loss of hydrogen bonds

To investigate the occurrence of HINT1 neuropathy in Norway, we analyzed a cohort of 748 patients with suspected peripheral neuropathy by next-generation sequencing targeting a panel of neuropathy genes. In two probands, belonging to seemingly unrelated families, we identified identical compound heterozygous variations in *HINT1*: the known pathogenic variant NM_005340.6:c.110G > C (p.Arg37Pro) [1] and a variant of unknown significance NM_005340.6:c.284G > A (p.Arg95Gln). The other 98 neuropathy genes included in the test panel (Additional file 1) were variation negative. Segregation analysis in family 1 confirmed that the variants were situated *in trans* (Fig. 1a). For patient 2 (P2), DNA from the parents was unavailable, yet cDNA analysis of RNA extracted from patient-derived lymphoblasts confirmed their location on different alleles (Fig. 1b). The two probands live in different states and a detailed genealogical investigation dating back to the great-grandparents of patient 1 (P1) and the grandparents of P2 did not reveal any common ancestors. To investigate if the novel c.284G > A variant in both probands originated from a single mutational event, we performed haplotype analysis as described [1]. Both patients shared a haplotype of 7.7 Mb (D5S1495-D5S2117) surrounding the c.284G > A variant, suggesting a founder effect. We also confirmed that they share the previously established haplotype surrounding the known c.110G > C founder allele (Fig. 1a) [1]. Both variants were reported in the Genome Aggregation Database (gnomAD v2.1.1) [12] but only in heterozygous state. Specifically, c.110G > C was found in 59:128,474 alleles in the European (non-Finnish) population, out of which 13 had Swedish origin. Its overall allele frequency was 0.0003018 (85:281,678 alleles). The c.284G > A variant was found in 5:113,666 alleles in the European (non-Finnish) population, out of which two were Swedish. In addition, the variant was reported in one out of 15,308 South-Asian individuals. To establish the frequency of the variations in the Norwegian population at large, we investigated 36,600 chromosomes collected from the four main genetic reference centers in Norway. In this dataset, we did not identify additional variants, suggesting that both the known Slavic c.110G > C founder variant and the novel c.284G > A variant have a low frequency in Norway. In two of the reference centers, allele counts below 4 or 5 were filtered out to ensure anonymity of personal data in compliance with the EU general data protection regulation (GDPR); therefore, the overall allele frequency of each variant in Norway was estimated between 0.00005464 (1:18,302)–0.00024587 (1:4,067).

Both patients underwent clinical and neurophysiological examinations, and the major findings are presented in Tables 1 and 2. P1 was born from an uneventful pregnancy with normal birth weight. He displayed mild motor delay with independent walking at 18 months of age and late language development. He had a physiotherapist in nursery school due to reduced balance and gait problems. When he was six years old, he was unsteady and ran slower than others of his age. In school, the patient tired quickly during sporting activities. He had always experienced difficulties opening and tightening caps. At his first neurological examination (age 12 years), he presented with reduced balance and steppage gait, strikingly thin lower legs, bilateral foot drop and decreased tendon reflexes in lower extremities. He reported considerable pain in thighs, lower legs and ankles after minimal exercise, pain in hands when writing and sudden episodes of muscle weakness in one leg. In addition, he displayed moderate contractures in elbow joints and suffered episodes of muscle stiffness and cramps, especially in the hands. Electroneurography (ENG) and electromyography (EMG) examinations at age 12 years revealed chronic motor and sensory axonal neuropathy with a predominating motor component (Table 2). At 13 years of age, he underwent bilateral Achilles tenotomy due to foot drop and pes cavovarus. As additional signs, the patient had urinary incontinence and was diagnosed with attention deficit hyperactivity disorder (ADHD), with reported mood and conduct problems. Neurological examination was repeated and Neuropathy Impairment Score (NIS) assessment was performed at age 14 years (Table 1). His parents and older sibling did not show clinical signs of neuropathy.

**Table 1:** Clinical characteristics of the patients with missense variants in *HINT1*

Patient	1	2
Age at onset (years)	6	20*
Disease duration	8	15
Age at investigation	14	35
Clinical characteristics		
Muscle wasting^1^		
Underarm	1	0
Hand	1	1
Thigh	0	1
Leg	2	2
Feet	2	2
Muscle weakness^NIS^		
Wrist extension	3	2/3^2^
Finger flexion	2	0
Finger spread	3	3
Thumb abduction	3/2^2^	3
Knee flexion	0	2
Knee extension	0	2
Ankle dorsiflexors	3.25	3.25
Ankle plantar flexors	2	3.25
Toe extensors	3.25	3.25
Toe flexors	3.25	3.25
Sensory loss		
Touch^1^		
Feet, leg	0	0
Arm, hand	0	0
Pain^1^		
Overarm	0	0
Hand, underarm	1	1
Thigh	0	1
Feet, leg	1	1
Vibration^1^		
Hand	0	2
2. finger	1	2
Knee	0	2
Ankle	2	2
1. metatarsal	2	2
1. toe	2	2
Proprioceptive^1^		
Toe	0	2
Reflexes^1^		
Biceps	2	1/2^2^
Triceps	1	1
Brachioradialis	1	1/2^2^
Patellar	2	2
Achilles	2	2
Romberg^1^	2	2
NIS score	70.5	82

*NIS* Neuropathy Impairment Score

^1^0 = normal; 1 = mild/moderate affected modality; 2 = severely affected modality

^2^Asymmetrical signs right/left

^*^Onset age reported by the patient

**Table 2:** Neurophysiology of the patients with missense variants in *HINT1*

	Sex	Age at		R/L	Motor nerves								Sensory nerves						EMG chronic denervation
		Onset (years)	Examination (years)		Median		Ulnar		Peroneal		Tibial		Median		Ulnar		Sural		
					CMAP	CV	CMAP	CV	CMAP	CV	CMAP	CV	SNAP	CV	SNAP	CV	SNAP	CV	
Normal values →					4.0	49.0	4.0	49.0	3.0	41.0	3.0	41.0	12.0	46.0	17.0	47.0	17.0	44.0	
Patient 1	♂	6	12	R	4.3	**43**	5.3	**46**	3.3	45	**0.2**	**A**	**10.6**	47	**3.0**	51	**4.4**	**40**	**Present**
Patient 2	♂	20*	32	R	5.6	**42.5**	**1.8**	**35.3**	**A**	**A**	**0.1**	**A**	**9.4**	**45.9**	**1.0**	**36.5**	**0.9**	**18.4**	**–**
			36	R	**1.3**	**36.1**	–	**–**	**A**	**A**	**A**	**A**	**2.8**	**45.3**	**–**	–	**2.9**	**26.6**	**Present**

*CMAP* compound motor action potential (mV), *SNAP* sensory nerve action potential (µV), *CV* conduction velocity (m/s), *A* absent evoked response, – not measured, *R/L* right/left

Abnormal values are represented in bold

^*^Onset age self-reported by the patient

P2 was referred at age 22 years for an electrophysiological examination due to gait disturbances, balance problems and foot drop. The patient reported an onset of his symptoms around age 20 years. He had earlier been healthy and active in sport but noted always to have had thin lower legs. ENG and EMG revealed chronic sensory and motor axonal neuropathy. He reported that the gait problems stopped six months after the initial examination and did not start again until about age 27–31 years. At age 32 years, he was re-examined due to two episodes of sudden muscle weakness and stiffness starting in one leg and spreading to the rest of the body. He also reported weaker legs than earlier and pain in lower extremities and hands. In addition, he experienced episodes of muscular stiffness and cramps in the hands. The neurological and electrophysiological evaluation revealed progression of the disease in lower extremities and hand involvement (Table 2). At age 35 years, NIS was performed (Table 1) and at age 36 years, ENG and EMG were repeated (Table 2). Neuromyotonia was not present on EMG in P1 and P2. In addition, both patients were tested clinically for delayed relaxation of the hand muscles after a prolonged voluntary contraction, P1 at age 14 years and P2 at age 35 years. No signs of neuromyotonia were observed in either of the patients.

The p.Arg95Gln substitution targets a conserved residue (Fig. 1c) and is predicted to affect HINT1 function (Polyphen-2v.2: probably damaging, score = 0.996; SIFTv.6.2.1: affecting protein function, score = 0.02; Mutation Taster.v.2: disease causing, score = 0.9999) [13, 14]. In silico modeling on the HINT1 crystal structure revealed that p.Arg95 is located at the dimer interface (Fig. 1d). The p.Arg95Gln substitution is predicted to abolish critical hydrogen bonds, leading to potential structural and/or functional destabilization effects.

**Fig. 2:**
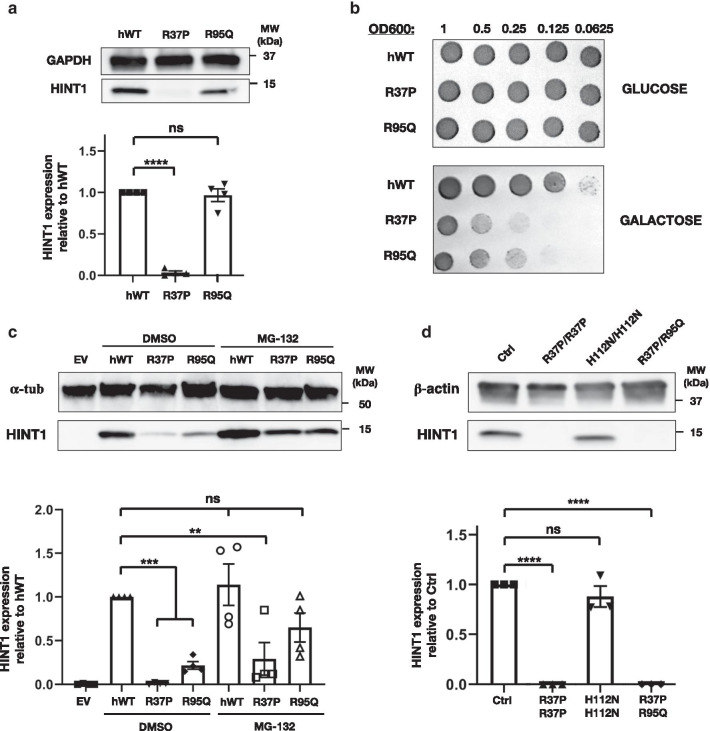
Functional characterization of the identified HINT1 variants. **a** Western blot analysis of protein extract from *HNT1*-deleted yeast strain expressing either the wild-type HINT1 or the disease-causing variants. Equal loading was validated with rabbit polyclonal anti-GAPDH antibody and relative HINT1 expression was normalized to hWT. Graph represents relative quantification of band intensities for four independent replicates. **b** Genetic complementation analysis in *HNT1-*deleted yeast strain performed by spot assay. Serial dilutions of the different yeast strains were spotted on minimal media without leucine, supplemented with either 2% glucose or 2% galactose, and incubated at 39 °C for 3 days. **c** Western blot analysis of protein extracts from HINT1-KO HeLa cell lines transiently transfected with either the wild-type HINT1 or the disease-causing variants. Graph represents relative quantification of band intensities for four independent replicates. **d** Western blot analysis of total protein extracts from HINT1 patients or control (Ctrl) lymphoblasts. Membranes were immunoblotted with polyclonal rabbit anti-human HINT1 antibody. Equal loading was validated with mouse monoclonal anti-β-actin and anti-α-tubulin antibodies and relative HINT1 expression was normalized to hWT and Ctrl expression. Graph represents relative quantification of band intensities for three independent replicates. Statistical one-way ANOVA analysis was performed. Bar charts are presented as means with standard error of the mean (s.e.m.)

To investigate the deleterious impact, we performed genetic complementation in yeast [1]. When expressing p.Arg95Gln HINT1 in an *HNT1* knockout (KO) yeast strain [7] expression levels were comparable to the wild-type human protein (Fig. 2a). Further growth analysis under stress conditions, however, revealed that the mutant protein did not rescue the *HNT1* deficiency (Fig. 2a), implicating it is functionally impaired. In contrast, the p.Arg37Pro protein was undetectable upon expression in yeast and did not complement the *HNT1* deficiency as established before (Fig. 2a, b) [1].

Next, we tested the detrimental effect of p.Arg95Gln in a mammalian model by transient transfection in a *HINT1* KO cell line. Unlike in the yeast model, in human cells immunoblotting of the p.Arg95Gln protein—similar to p.Arg37Pro HINT1—showed a considerably reduced expression compared to wild-type HINT1. Cell treatment with the MG-132 inhibitor caused accumulation of both p.Arg95Gln and p.Arg37Pro proteins, confirming their degradation is mediated by the proteasome (Fig. 2c). Our findings in the human KO cells were supported by immunoblot analysis in lymphoblasts from P2, where we did not detect any HINT1 in total protein extracts (Fig. 2d), confirming the destabilizing effect of both substitutions in human cells and establishing HINT1 deficiency as the cause of the axonal neuropathy.

## Discussion

This is the first report of HINT1-neuropathy in Northern Europe. In two seemingly unrelated Norwegian individuals, identical compound heterozygous *HINT1* substitutions (p.Arg37Pro/p.Arg95Gln) were identified. Both patients had a severe axonal neuropathy (NIS = 70.5 and 82). Consistent with NMAN symptomatology, there was predominant motor involvement [2]. Despite being described as a diagnostic hallmark, neuromyotonia is absent from 20 to 30% of patients and is often underdiagnosed [2, 6]. After establishing genetic diagnosis, both patients were tested clinically for neuromyotonia and EMG was repeated, yet no signs of neuromyotonia were detected. Nevertheless, P1 had contractures and both patients displayed episodes of muscular cramps in the hands, which can be perceived as subtle signs of neuromyotonia [2].

Both patients complained about pain in hands and lower extremities, a symptom that so far has not been documented for HINT1-neuropathy. In patients with peripheral neuropathy, pain is a relatively frequent but underreported symptom [15]. Although the causal association between pain and HINT1 neuropathy is uncertain, studies in *HINT1* KO mice showed a link with nociception and demonstrated a modulatory effect on the cannabinoid pathway [16, 17]. Moreover, P1 had non-classical symptoms suggesting CNS involvement, ranging from late language development to social behavioral alterations. Such rare symptoms have been reported in only a few NMAN patients. Speech difficulties were described in two patients with homozygous p.Arg37Pro variants [9, 11]. One of them and another recently reported patient [10] also displayed a mood and severe conduct disorder. Taken together, our findings and the previous case reports indicate that CNS involvement may be considered in the differential diagnosis of NMAN, but detailed genotype–phenotype correlation studies on larger patient cohorts are needed to confirm a causal relation.

Haplotype analysis confirmed that the c.284G > A transition in the two Norwegian patients resulted from a single mutational event. In online databases, five additional c.284G > A alleles were observed in European carriers, of which at least two were from Northern Europe as well. It would be interesting to examine the haplotype surrounding those alleles to determine if this substitution represents an additional rare founder variant in *HINT1*. In addition, our findings expand the geographical distribution of the p.Arg37Pro disease haplotype to Northern Europe and provide further evidence that this ancient Slavic allele has an unequal distribution in Europe [2].

Immunoblot analysis showed absence of HINT1 protein in patient-derived lymphoblasts, in line with earlier studies where 80% of disease-causing substitutions lead to protein instability and subsequent proteasome-mediated degradation [1]. The p.Arg95Gln substitution is localized in a small segment (Tyr94-Glu100) at the dimer interface where multiple NMAN-causing variants cluster: p.Gly93Asp, p.Tyr94Cys, p.Val97Met. [1, 4, 5, 18] The p.Arg95Gln variation could abolish crucial hydrogen bonds within and between the monomers, leading to quaternary structure destabilization and a misfolded protein that is degraded by the proteasome. Likewise, amino acid substitutions in the same segment, as well as other NMAN variants impair dimerization and enzymatic activity in vitro [19]. Our functional studies in patient-derived lymphoblasts and KO cells provide the first in vivo evidence that a substitution in that specific region of the dimer interface causes HINT1 protein destabilization.

Because HINT1 functions as a homodimer, this poses the question whether the formation of heterodimers might cause additive destabilization effects. Notably, most of the variants causing HINT1 neuropathy occur at a low frequency and, similarly to this report, often patients are compound heterozygous. In fact, only p.Arg37Pro, p.His112Asn and p.Cys38Arg have been found in homozygous state and only for the first two has it been possible to assess their effect on the protein stability independently of any other variant [1]. Therefore, we expressed each of the two compound heterozygous variants individually in *HINT1* KO cells. Upon transient overexpression, traces of both proteins were detected via immunoblotting; yet p.Arg95Gln appeared to be more stable compared to p.Arg37Pro protein under the same conditions. Following proteasome inhibition, both mutant proteins accumulated over time (Fig. 2c), indicating that both are unstable and prone to degradation in human cells. Contrastingly, in yeast, p.Arg95Gln protein was not fully degraded, allowing us to assess its functionality. The lack of genetic complementation demonstrated that, unlike other destabilizing NMAN-variants that preserve the catalytic activity, e.g. Cys84Arg [19], the produced p.Arg95Gln protein is inactive. This finding has important implications for patient stratification in future therapeutic strategies, as p.Arg95Gln-carriers belong to a subgroup of patients who would not benefit from treatment with a HINT1-stabilizing compound.

## Conclusions

This study represents the first analysis of HINT1 neuropathy in Norway, where we identified a rare novel allele (p.Arg95Gln) and investigated the frequency of the most common HINT1 allele (p.Arg37Pro) in a large population sample from Norway. Functional characterization in human and yeast cell models provided mechanistic insights on how the newly reported substitution leads to loss of HINT1 function. The patients displayed motor-predominant peripheral neuropathy of variable onset, associated with pain in the four limbs and neuro-psychiatric symptoms. Our findings expand the genetic epidemiology of *HINT1*-related disorders.

## Methods

### Patients and evaluation

The patient cohort comprised 748 individuals with suspected peripheral neuropathy, who had previously tested negative for duplications or deletions in *PMP22*. The mean age at referral was 50.5 years old and 11% of patients (n = 84) were below 20 years of age at the time of testing.

The two probands underwent detailed neurologic examination (performed by G.J.B. and L.V.B.). Three asymptomatic family members (the parents and unaffected sibling of P1) were also evaluated. Standard nerve conduction studies and electromyography were performed following standard procedure. Information on additional clinical exams was collected from electronic hospital records.

### Next-generation sequencing

NGS was performed using an in-house designed panel containing the coding exons and flanking intronic sequences of 99 neuromuscular disease-associated genes (Additional file 1) according to the Illumina’s Nextera standard protocol (Illumina Inc., San Diego, USA) and by sequencing on an Illumina NextSeq 500 following standard procedures. Reads were mapped to the reference sequence (GRCh37/hg19) by Burrows-Wheeler Alignment tool [20]. The Genome Analysis ToolKit was used for base quality score recalibration, indel realignment, duplicate removal, and SNP and INDEL discovery [21–23]. Variants were annotated with the ANNOVAR software tool [24]. Bioinformatic filtering was done using the Filtus software [25]. Variant verification and segregation analysis were performed by Sanger sequencing.

### Haplotype analysis

Haplotype sharing analysis for both variants was performed using a previously described panel of STR and SNP markers [1]. STR genotyping was done by capillary electrophoresis of fluorescently labeled amplicons containing the marker region (3730xl DNA analyzer, Applied Biosystems, Foster City, CA, USA). SNP genotyping was performed by Sanger sequencing.

### RNA isolation and cDNA analysis

Total RNA was isolated from lymphoblasts using the Universal RNA kit (Roboklon, Berlin, GE) and treated with the Turbo DNA-free kit (Ambion, Austin, TX, USA) to remove residual gDNA. RNA was transcribed to single strand cDNA with the iScript cDNA advanced synthesis kit (Bio-Rad, Hercules, CA, USA). HINT1 cDNA was amplified using HINT1-specific primers with overhangs containing restriction sites for EcoRI (forward primer) and HindIII (reverse primer) and introduced into a pUC19 plasmid after double digestion with FastDigest-EcoRI and FastDigest-HindIII (ThermoFisher Scientific, Waltham, MA, USA) followed by ligation with T7 DNA ligase (Enzymatics, Beverly, MA, USA). Products were transformed into E. coli Mach1 chemically competent cells (ThermoFisher Scientific, Waltham, MA, USA) and plated on ampicillin agar. Positive colonies were picked and grown overnight in 5 ml LB broth supplemented with 100 µg/ml ampicillin. Cells were harvested and plasmid was purified using the NucleoSpin Plasmid EasyPure kit (Macharey-Nagel, Bethlehem, PA, USA). Phasing of the variants was performed by Sanger sequencing of individual clones.

### In silico modeling

The modeling of the new p.Arg95Gln variant was performed using Chimera v.1.14 on the HINT1 (PDB ID: 5KLZ) crystal structure. The position of the new amino acid change within the structure was estimated using the Rotamers tool with standard parameters.

### HINT1 expression plasmids

Yeast expression plasmids carrying human HINT1 (pAG415-HINT1-hWT & pAG415-HINT1-Arg37Pro) were generated in a previous study [1]. Mammalian expression plasmid carrying human HINT1 (pCAGGS-HINT1-hWT) was created at the VIB Protein Service Facility (UGent, Ghent, BE). The different HINT1 variants were introduced with site directed mutagenesis using KAPA HiFi DNA polymerase (Roche Diagnostics, Basel, CH). After overnight DpnI digestion (New England Biolabs, Ipswich, MA) products were transformed into *E. coli* Mach1 chemically competent cells (ThermoFisher Scientific, Waltham, MA, USA) and validation of the correct incorporation of the missense variant was done by Sanger sequencing of the purified plasmid.

### Yeast strain and transformation

*S. cerevisiae* strain BY8-5c [7] (MATα *ura3-52 his3Δ200 trp1Δ901 lys2-801 suc2-Δ9 leu2-3,112 hnt1Δ::URA3*) was provided by Dr. Brenner, University of Iowa, USA. Yeast cells were cultured in rich medium (YPD). Transformation of BY8-5c with the pAG415GPD expression plasmids carrying one of the HINT1 variants or the human wild-type was done with the LiAc/SS carrier DNA/PEG method [26]. Positive clones were selected in minimal medium without Leucine (SD-Leu) supplemented with 2% glucose.

### Spot assay

Pre-cultures of the different yeast clones were grown overnight in SD-Leu supplemented with glucose. Absorbance was measured and adjusted to an optical density of OD_600nm_ = 1. Serial dilutions of each culture were spotted in 5ul drops on SD-Leu agar plates supplemented either with 2% glucose or 2% galactose. Plates were incubated for three days at 39ºC.

### Cell line establishment and culture

Peripheral blood lymphocytes of P2 were isolated using a Ficol Paque gradient and subsequently transformed with Epstein-Barr virus. After a two-hour incubation at 37 °C, cells were centrifuged and re-suspended in RPMI complete medium (Invitrogen, Carlsbad, CA, USA) supplemented with 1% phytohaemagglutinin. Cells were seeded on a 24-well plate and incubated at 37 °C and 5% CO_2_ for three days. After establishment, lymphoblastoid cells were grown in RPMI complete medium containing 15% fetal bovine serum (FBS, Gibco, Waltham, MA, USA), 1% sodium pyruvate, 1% L-Glutamine (Gibco, Waltham, MA, USA) and 1% penicillin/streptomycin (Gibco, Waltham, MA, USA).

*HINT1* HeLa KO cell lines were created using CRISPR/Cas9 mediated gene editing according to the standard protocol [27]. The single guide RNA (sgRNA) sequence conferring target sequence specificity to the CRISPR/Cas9 system was designed using the sgRNA Designer (Broad Institute). The sgRNA with the highest combined rank was selected (CTTTGGGAAGATCATCCGCA) targeting the first exon of HINT1 with a predicted on-target efficacy of 0.6315. Complete KO of HINT1 was validated on the genomic level by Sanger sequencing and on the protein level by immunoblotting.

HeLa cells were grown in high-glucose DMEM (Gibco, Waltham, MA, USA) supplemented with 10% heat-inactivated fetal bovine serum (FBS, Gibco, Waltham, MA, USA), 1% glutamine (Gibco, Waltham, MA, USA) and 1% penicillin/streptomycin (Gibco, Waltham, MA, USA). Cells were cultured at 37ºC and 5% CO_2_ in a humidified atmosphere.

### Cell transfection and MG-132 treatment

HeLa cells were transiently transfected using polyethylenimine (PEI) MW25000 (Polysciences, Warrington, PA, USA). Briefly, cells were seeded out in a 6-well plate the day before transfection in complete medium without antibiotics. At 70–80% confluency, cells were transfected with PEI and 250 ng of the pCAGGS vector carrying either one of the HINT1 variants or the human wild-type. A pCAGGS-MBP was used as an empty vector control. 24 h after transfection cells were treated overnight with 20 µM MG-132 (Sigma Aldrich, San Luis, MO, USA) or solvent only (DMSO, Merk, Kenilworth, NJ, USA). Cells were harvested the next day by trypsinization.

### Immunoblotting

Human cells were lysed in RIPA lysis buffer (20 mM Tris–HCl pH = 7.4; 150 mM NaCl; 0.1% Nonidet P-40; 0.5% sodium deoxycholate; 0.1% sodium dodecyl sulfate) supplemented with Halt™ Protease Inhibitor Cocktail (ThermoFisher Scientific, Waltham, MA, USA). Protein concentration was determined with the Pierce BCA protein assay kit (ThermoFisher Scientific, Waltham, MA, USA) and adjusted to 20 µg per sample. Lysates were boiled for five minutes in reducing Laemmli sample buffer (Bio-Rad, Hercules, CA, USA) supplemented with 100 mM 1.4-Dithiothreitol (DTT).

Yeast proteins were extracted following a previously published protocol [28]. Briefly, cells were collected before stationary phase (OD_600nm_ = 1) by centrifugation. Then cells were washed first with 2.0 M LiAc and then 0.4 M NaOH for 5 min on ice. Cells were finally boiled for five min in Laemmli sample buffer (Bio-Rad, Hercules, CA, USA) supplemented with 100 mM DTT.

Proteins were separated in 4–15% Mini-PROTEAN® TGX Stain Free™ Protein gels (Bio-Rad, Hercules, CA, USA) and transferred to a nitro-cellulose membrane (Hybond™-P, GE Healthcare, Chicago, IL, USA) using the semi-dry Trans-Blot® Turbo™ Transfer System (Bio-Rad, Hercules, CA, USA). Membranes were blocked for an hour at room temperature with 5% milk powder diluted in PBS supplemented with 0.1% Tween-20 and then incubated with primary antibody overnight at 4ºC and one hour with a secondary horseradish peroxidase-conjugated antibody at room temperature. Blots were developed with Enhanced Chemiluminiscence ECL Plus™ (ThermoFisher Scientific, Waltham, MA, USA) and imaged with ImageQuant™ LAS 4000 (GE Healthcare, Chicago, IL, USA).

The antibodies used in this study were: polyclonal rabbit anti-human HINT1 antibody (1:1000, Sigma, San Luis, MO, USA), and to demonstrate equal loading: mouse monoclonal anti-β-actin antibody (1:5000, Sigma, San Luis, MO, USA), mouse polyclonal anti-α-tubulin antibody (1:5000, Abcam, Cambridge, UK) or rabbit polyclonal anti-GAPDH antibody (1:20,000, GeneTex, Irvine, CA, USA).

## Supplementary Information

**Additional file 1.** Gene panel. List of the 99 neuromuscular disease-associated genes included in the in-house designed panel.

## Data Availability

All data generated or analyzed during this study are included in this published article.

## Abbreviations

ADHD: Attention deficit hyperactivity disorder
AMP: Adenosine monophosphate
BCA: Bicinchonic acid
CAS9: CRISPR associated protein 9
CNS: Central nervous system
CRISPR: Clustered regularly interspaced short palindromic repeats
Ctrl: Control
DMSO: Dimethyl sulfoxide
EMG: Electromyography
ENG: Electroneurography
FBS: Fetal bovine serum
GAPDH: Glyceraldehyde-3-phosphate dehydrogenase
GDPR: General data protection regulation
gnomAD: Genome Aggregation Database
HINT1: Histidine Triad Nucleotide Binding protein 1
HIT: Hisitidine triad
HNT1: Hit family protein 1
KO: Knockout
LB: Luria broth
LiAc: Lithium acetate
MBP: Maltose-binding protein
NCV: Nerve conduction velocity
NGS: Next-generation Sequencing
NIS: Neuropathy impairment score
NMAN: Neuromyotonia, axonal neuropathy
PBS: Phosphate buffered saline
PDB: Protein Data Bank
PEG: Polyethylene glycol
PEI: Polyethylamine
SD-Leu: Synthetic defined medium without leucine
sgRNA: Single guided RNA
SNP: Single nucleotide polymorphism
STR: Single tandem repeat
SUMO1: Small ubiquitin-like modifier 1
YPD: Yeast extract peptone dextrose
