# Supplementary material for: HINT1 neuropathy in Norway: clinical, genetic and functional profiling
Source: Orphanet J Rare Dis. 2021 Mar 4;16:116. doi: 10.1186/s13023-021-01746-z (PMC7934415; doi:10.1186/s13023-021-01746-z)
Supplement: Supplementary file 1 — Additional file 1. Gene panel. List of the 99 neuromuscular disease-associated genes included in the in-house designed panel. [file 13023_2021_1746_MOESM1_ESM.docx]

**Ninety-nine CMT-related genes included in the NGS-panel**.

*AARS, AIFM1, ARHGEF10, ATL1, ATL3, ATP7A, BICD2, BSCL2, CCT5, COX6A1, CTDP1, DCAF8, DCTN1, DCTN2, DHTKD1, DNAJB2, DNM2, DNMT1, DST, DYNC1H1, EGR2, FAM134B, FBLN5, FBXO38, FGD4, FIG4, GAN, GARS, GDAP1, GJB1, GJB3, GNB4, HARS, HINT1, HK1, HOXD10, HSPB1, HSPB3, HSPB8, IGHMBP2, IKBKAP, INF2, KARS, KIF1A, KIF1B, KIF5A, LAMA2, LITAF, LMNA, LRSAM1, MARS, MED25, MFN2, MME, MORC2, MPZ, MTMR2, MYH14, NAGLU, NDRG1, NEFL, NGF, NTRK1, PDK3, PLA2G6, PLEKHG5, PMP22, POLG, PRNP, PRPS1, PRX, RAB7A, REEP1, SBF1, SBF2, SCN11A, SCN9A, SEPT9, SETX, SH3TC2, SLC12A6, SLC25A46, SLC5A7, SOD1, SOX10, SPG11, SPTLC1, SPTLC2, SURF1, TDP1, TFG, TRIM2, TRPV4, TUBB3, VAPB, VCP, VRK1, WNK1, YARS.*
